# Heart Rate Reduction and the Prognosis of Heart Failure Focused on Ivabradine

**DOI:** 10.3390/jcm14041074

**Published:** 2025-02-07

**Authors:** Shunsuke Kiuchi, Takanori Ikeda

**Affiliations:** Department of Cardiovascular Medicine, Toho University Faculty of Medicine, 6-11-1 Omori-nishi, Ota-ku, Tokyo 143-8541, Japan; takanori.ikeda@med.toho-u.ac.jp

**Keywords:** heart failure, heart rate, ivabradine, exercise tolerance

## Abstract

Cardioprotective medications referred to as the fantastic four are used to treat heart failure (HF). Additionally, ivabradine can also be used if the heart rate (HR) is elevated. An elevated HR is a prognostic factor in HF patients, as well as in the general population. In both HF with reduced ejection fraction (HFrEF) and HF with preserved ejection fraction (HFpEF), an elevated HR is associated with all-cause mortality, whereas cardiovascular death is only associated with the former. In addition, previous clinical trials revealed that ivabradine was useful only in HFrEF but not in HFpEF. Therefore, ivabradine is indicated for patients only with HFrEF. Moreover, ivabradine increases the stroke volume by ensuring an effective diastolic time as a result of the decreased HR. Including this effect, the introduction of ivabradine allowed for the discontinuation of dobutamine infusion used in HF patients and the uptitration of β-blockers in other reports. Additionally, ivabradine improves exercise tolerance and the subjective symptoms of HF. However, the effects of ivabradine on exercise tolerance remain poorly understood, and prospective clinical trials are underway. While these beneficial effects have been reported, side effects such as photopsia and atrial fibrillation have also been reported. It is important to use ivabradine appropriately in conjunction with standard HF treatment, including quadruple therapy.

## 1. Introduction

In Japan, the number of patients with heart failure (HF) continues to increase and is expected to reach approximately 1.3 million in 2030 [1]. In an analogy with infectious diseases, this is referred to as the HF pandemic and is an important problem worldwide [2]. In Japan, where the population is aging, the increase in the number of elderly patients afflicted with HF is expected to continue even after 2030 [3]. Thus, the number of hospitalized HF patients is anticipated to increase even after 2030 and is predicted to peak in 2040 [4]. Moreover, it has been reported that the hospitalization of heart failure patients is repeated, thus increasing economic burden [5]. Therefore, in an HF pandemic, it is important not only to improve HF prognosis but also to prevent the rehospitalization of patients with HF. In HF treatment aimed at improving prognosis, quadruple therapy, which includes angiotensin-converting enzyme inhibitors (ACE-Is)/angiotensin II type 1a receptor blockers (ARBs)/angiotensin receptor neprilysin inhibitors (ARNIs), β-blockers (BBs), mineralocorticoid antagonists (MRAs), and sodium glucose cotransporter-2 inhibitors (SGLT2is), has been recommended for HF with reduced ejection fraction (EF) (HFrEF) [6,7]. Conversely, cardioprotective medication, which has insurance coverage for HF preserved EF (HFpEF), includes SGLT2is only. SGLT2is focus on neurohumoral factors such as the renin–angiotensin–aldosterone system or cardiac sympathetic nerve activity or new medications for the treatment of HF, with various mechanisms. Ivabradine has also been launched as a new therapeutic medication for HF, and the relationship between HF and heart rate (HR) has been highlighted. In this review, we will discuss the management of HR in HF, focusing on ivabradine.

## 2. The Relationship Between HR and HF

An elevated HR reflects the body’s metabolic demands, and a high HR is a prognostic factor in many animal species [8]. In humans, a general population study reported that elevated HR is associated with disease prognosis [9], which is similar when applied to HF [10]. Increased HR leads to HF exacerbation, resulting in many complications, including atherosclerotic disease [11]. Conversely, it has been suggested that the HR in HF differs depending on the cardiac rhythm and EF. A higher HR is associated with increased mortality in sinus rhythm and poor prognosis in atrial fibrillation if the HR is excessively elevated [12]. According to the EF classification, a high HR is associated with all-cause mortality both in HFrEF and HFpEF; however, cardiovascular death is associated with a high HR only in HFrEF [13]. Including this result, ivabradine is only indicated for HFrEF. The minute cardiac output can be calculated by multiplying stroke volume (SV) by HR. Therefore, in response to a decrease in SV, the heart responds by increasing the HR to maintain hemodynamics. Additionally, an increased HR also reflects increased cardiac sympathetic activity in patients with HF [14], and this sympathetic overactivity is associated with a poor prognosis in HF [15]. A reduction in HR in HF contributes to improvement in prognosis, which has been achieved through the administration of a BB and ivabradine as reported in one study [16,17]. However, if the former relationship (SV and HR) is more strongly involved, the relationship between HR and prognosis may be weak, and the effect may differ depending on the pathology of HF. Attempts have also been made to assess the optimal HR for HF based on echocardiography [18,19]. HR in HF is not only a surrogate marker but also a therapeutic target [20].

## 3. Effects of Ivabradine on HF Prognosis

Ivabradine is a medication that inhibits the hyperpolarization-activated cyclic nucleotide-gated (HCN) channel. The HCN channel was classified into four subunits [21], and in the heart, ivabradine has an HR-lowering effect through the suppression of the hyperpolarization-activated cation current (If) via the inhibition of the HCN4 channel expressed in the sinus node [22]. Ivabradine reduces HR without affecting the systolic blood pressure (BP), cardiac conduction, or contractility [23]. For HFrEF, a Systolic Heart Failure Treatment with the IF Inhibitor Ivabradine Trial (SHIFT) was conducted in Europe [24]. A total of 6505 patients with chronic HF (excluding Japanese patients) with sinus rhythm, a resting HR > 70 bpm, and a left ventricular (LV) EF ≤ 35% were evaluated. This study examined the effects of administering ivabradine to patients who had been receiving standard HF medication, such as the maximally tolerated BBs, ACE-Is, ARBs, or MRAs, and had been in a stable condition for more than four weeks. The primary outcome of cardiovascular death or hospitalization due to worsening HF was significantly reduced by 18% in the ivabradine group (ivabradine 24.5% vs. placebo 28.7%; hazard ratio 0.82, 95% CI: 0.75–0.90, *p* < 0.0001). The risk of hospitalization due to worsening HF was reduced by 26% (ivabradine 16% vs. placebo 21%; hazard ratio 0.74, 95% CI: 0.66–0.83, *p* < 0.0001). Based on these results, a Japanese SHIFT phase III study (J-SHIFT) was conducted in Japan. In the J-SHIFT study, LVEF ≤ 35% was the same as that in the SHIFT study; however, resting HR was defined as >75 bpm, and 254 patients with chronic HF were enrolled [17]. The results were similar to those in the SHIFT trial, with a 33% risk reduction in the primary endpoint (ivabradine 20.5% vs. placebo 29.1%; hazard ratio 0.67, 95% CI: 0.40–1.11, *p* < 0.1179). From the results of these studies, the introduction of ivabradine is recommended for HFrEF patients who had been administered optimal medical treatment for HF from guidelines, had sinus rhythm, and had a resting HR > 75 bpm [7]. However, the effects of ivabradine remain uncertain in HFpEF [25]. Data from previous studies are summarized in Table 1. Among these studies, the prEserveD left ventricular ejectIon fraction chronic heart Failure with ivabradine studY (EDIFY), in which a large number of patients were enrolled, is well known [26]. A total of 179 HFpEF patients with sinus rhythm, HR ≥ 70 bpm, N-terminal pro-brain natriuretic peptide (NT-pro BNP) levels ≥ 220 pg/mL (BNP ≥ 80 pg/mL), and LVEF ≥ 45% were enrolled in the EDIFY study. In these patients, the effects of ivabradine administration on diastolic function determined by transthoracic echocardiography, distance in the 6 min walking test, and HF biomarker (NT-pro BNP) were evaluated. Ivabradine did not affect these findings. There was also no difference in adverse events, including HF, indicating no difference in prognosis. In a study conducted in Japanese subjects, ivabradine also did not improve left ventricular diastolic dysfunction [27]. On the other hand, it has been also reported that ivabradine improved LV diastolic function evaluated using two-dimensional and tissue Doppler echocardiographic patterns [28]; thus, there are no consistent reports on the effects of ivabradine on LV diastolic dysfunction. From these study results for HFpEF, ivabradine is currently not indicated for HFpEF.

## 4. Pathophysiology of HFpEF and Use of Ivabradine

HF is diagnosed according to the Framingham criteria and some guidelines [31,32] and is classified as HFpEF if LV systolic function is maintained. According to Japanese guidelines [7], HFpEF is diagnosed when EF is 50% or higher. The HFpEF assessment score includes E/e’ as measured by Doppler echocardiography [33], indicating LV diastolic dysfunction; however, the guidelines do not include the degree of LV diastolic dysfunction in the diagnosis of HFpEF. This indicates that HFpEF includes many pathological conditions in addition to LV diastolic dysfunction. Currently, only SGLT2 inhibitors are useful for HFpEF. Additionally, SGLT receptors are not present in the heart, and SGLT receptor 2 is expressed only in the kidney. Therefore, various mechanisms have been shown to be involved in the positive effects of SGLT2 inhibitors on heart failure [34]. Since HFpEF includes many pathological conditions, SGLT2 inhibitors with various mechanisms may be effective. One of the various mechanisms of HFpEF is thought to be vascular insufficiency (arteriosclerosis). During LV systole, about 60% of the blood ejected from the LV flows to the peripheral circulation, whereas the remaining 40% is stored in the arteries, which are elastic. And the stored blood flows to the periphery organization during LV diastole. This is called the Windkessel effect, and it prevents excessive afterload enhancement [35]. Taking these mechanisms into account, in the acute phase of HF, patients should be classified by clinical scenario (CS) classification, and the initial treatment is recommended to be started within 10 min, according to the Japanese guidelines [7]. The CS classification is based on BP [36], and it has been reported that patients that are categorized in CS1, who have high BP, also have vascular failure [37]. Ivabradine has also been reported to increase arterial compliance, thereby reducing left ventricular afterload [13].

**Figure 1:**
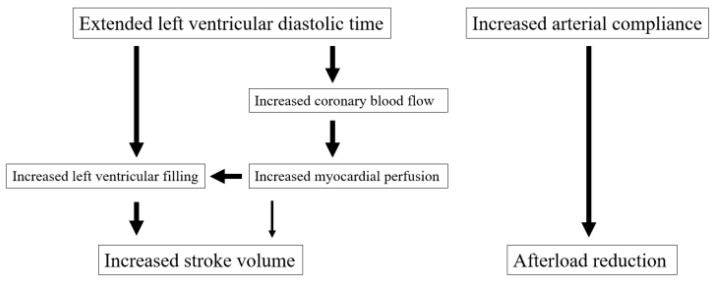
These mechanisms underlie the usefulness of ivabradine for HFpEF.

Additionally, the degree of LV diastolic dysfunction is also naturally involved in the pathogenesis of HFpEF. When LV diastolic dysfunction is strongly involved in the pathogenesis of HFpEF, a reduction in HR leads to the extension of LV diastolic time; thus, HR may have a large impact on the prognosis of HFpEF. A previous study had reported that an elevated HR was associated with an increase in cardiovascular mortality in HFpEF compared to HFrEF [38]. These mechanisms underlying the usefulness of ivabradine for HFpEF are summarized in Figure 1 (also effective for HFrEF). However, no prospective studies have been conducted on the impact of HR control on prognosis in HFpEF. In two studies on HFrEF (DAPA and EMPEROR-reduced studies), the addition of an SGLT2 inhibitor to standard treatment reduced the risk by approximately 25% [39]. On the other hand, in the SHIFT and J-SHIFT studies, which also targeted HFrEF, the risk reduction was approximately 30%, demonstrating efficacy equivalent to or greater than that of SGLT2 inhibitors [17,23]. In a pooled analysis of the DELIVER and EMPEROR-Preserved studies, the addition of an SGLT2 inhibitor to HFpEF patients receiving standard treatment reduced risk by approximately 20% [40]. From these findings, it is required that a group (phenotype group) with HFpEF on which ivabradine has a pronounced effect is identified. In addition, HFpEF is often accompanied by atrial fibrillation (AF); it has been reported that lowering HR is ineffective when AF is present. Future studies are needed to clarify the role of HR in the prognosis of HFpEF; however, ivabradine has also been reported to improve subjective symptoms [41]. Improvement in exercise tolerance also contributes to improvement in subjective symptoms, and ivabradine administration improves exercise tolerance in HFpEF patients [30]. On the other hand, there are also reports that the administration of ivabradine has no effect on exercise tolerance [29], and the research results are diverse. In many reports, patient backgrounds, such as the number of participants, target heart disease, type of HF based on EF, and types and doses of concomitant medications, differ [42,43,44] (Table 2). Studies on the ability of ivabradine to respond to exercise in HFrEF patients with tachycardia who are receiving standard therapy are also underway [45].

## 5. Cardiac Effects of Ivabradine Administration

**Figure 2:**
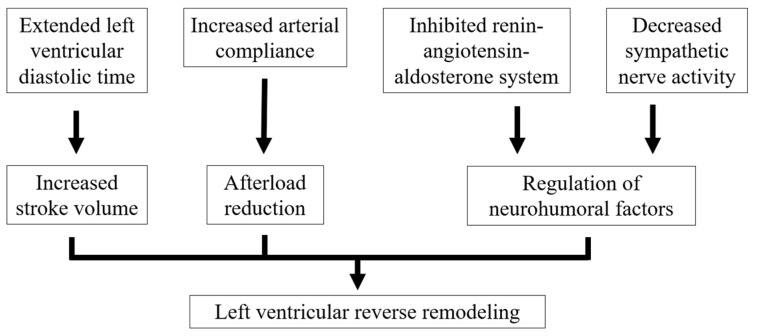
The efficacy of ivabradine in LV remodeling in HF.

The mechanisms of ivabradine that are involved in the improvement in HF symptoms and the prognosis of HF remain poorly understood. It has been reported that recovered LVEF is associated with better prognosis in HFrEF [46]; thus, one of the important therapeutic goals is the reversal of LV remodeling in HFrEF. Ivabradine has also been reported to be associated with LV reverse remodeling [17]. The administration of ivabradine to patients with HFrEF and coronary artery disease has been shown to reduce the LV end-systolic volume index and improve EF [47]. Ivabradine also inhibits dopamine-induced LV remodeling in HF after myocardial infarction [48]. Quadruple therapy for HFrEF (ACE-Is, ARBs, ARNIs, MRAs, BBs, SGLT2is) is also associated with LV reverse remodeling; however, these medications are often difficult to introduce because of their antihypertensive effects. In contrast, ivabradine is relatively easy to introduce because it does not affect BP nor impose negative inotropic effects. Figure 2 summarizes the efficacy of ivabradine in LV remodeling in HFrEF. Regarding ivabradine, in addition to manuscripts showing that it inhibits the renin–angiotensin–aldosterone system, there are also manuscripts showing that it does not [49,50]. In recent years, ARNIs have been used more frequently instead of ACE-Is and ARBs for the treatment of HF. The combination of ARNIs and ivabradine has also been shown to be effective for HFrEF patients with tachycardia, and simultaneous use may be better than sequential use [51]. The combination of ACEIs, BBs, MRAs, and ivabradine has also been shown to reduce rehospitalization due to the worsening of HF for HFrEF, and such combinations are also useful [52]. However, it is currently unclear which combinations of ivabradine with each medication are the most effective. The guidelines recommend the use of ivabradine in patients with sinus rhythm tachycardia after standard medication for HFrEF. We hope that the effectiveness of ivabradine for HFrEF and HFpEF and better combinations of ivabradine and other medications will become clearer as more studies are conducted.

## 6. β-Blockers and Ivabradine

Introducing a method of using BB-based cardioprotective medications for HFrEF has also been proposed [53], and it has also been shown to be useful to use ivabradine in combination with BBs. Even in J-SHIFT, in which the administration of the maximum dose of BBs was necessitated, only 20% of subjects were able to increase the dose to the maximum, and approximately 50% or more subjects were given a dose less than half of the maximum dose [17]. Because ivabradine has no effect on BP, it has also no effect on cardiac output (CO), which is a component of BP [23]. CO is maintained by compensating for the decrease in HR with ivabradine by increasing stroke volume. The increase in SV is thought to result from the extension of effective diastolic time with ivabradine [54]. BBs also decrease HR and extend diastolic time. However, isovolumic ventricular relaxation is also inhibited, offsetting some of this benefit in terms of the diastolic pressure–time integral. From the above results, it is suggested that the combination of ivabradine and BBs facilitates uptitration to the target BB dose [55]. In addition, in HF patients in serious condition, it can be difficult to discontinue dobutamine; however, a case of a patient has also been reported in which dobutamine was successfully discontinued with the concomitant use of ivabradine [56]. Based on the above, early initiation and the early combination therapy of ivabradine during hospitalization are expected to be useful. In HFrEF patients, the combination of ivabradine with BBs early in hospitalization improved functional and clinical parameters, including LVEF, 4 months later [57]. On the other hand, the combination did not improve the prognosis of HF 4 months or 1 year later [57,58]. A prospective clinical study on acute HF (SHIFT-AHF trial) is also being conducted, and the results are expected to be published [59]. Ivabradine and BBs have different effects on blood vessels. BBs have α-adrenergic coronary vasoconstriction. On the other hand, ivabradine not only does not retain this effect but also maintains effective diastolic time, thereby maintaining coronary blood flow [60]. Therefore, combined treatment with ivabradine and BBs improves myocardial perfusion through these mechanisms both at rest and during exercise [61]. Due to these effects, one study investigating the efficacy of BBs alone and a combination therapy of BBs and ivabradine in HF showed that although BBs alone led to a lower HR, the combination therapy of BBs and ivabradine led to a significantly better prognosis [62].

## 7. Safety Profile of Ivabradine

Photopsia is a well-known side effect of ivabradine; however, bradycardia should also be noted as a side effect. HCN channels are collections of four identical or distinct alpha subunits. There are four different genes in HCN subunits (1–4). Ivabradine exerts its effects on HR through HCN4 channel inhibition [22]; however, it has no selectivity for HCN channels. HCN channels are highly expressed in the sinoatrial node in the heart; however, they are also expressed to a small extent in the myocardium and in other tissues outside the heart, such as the optic nerve. The inhibition of HCN1 channels in the photoreceptors of the optic nerve attenuates light stimuli, resulting in photopsia. This was observed in approximately 2.7% and 6.3% participants in the SHIFT and J-SHIFT studies [17,23], respectively; however, owing to its transient nature, the symptoms disappeared when ivabradine was discontinued [63]. Moreover, the expression of HCN channels in ventricular myocytes is increased in HF. In this situation, the depolarization of diastolic cardiomyocytes and a reduction in the amplitude of the action potential overshoot occur, leading to premature ventricular contractions and ventricular arrhythmias. The use of ivabradine has been reported to reduce ventricular arrhythmias in HFrEF [64]; however, clinical evidence is limited, and the effects of ivabradine on ventricular arrhythmias in clinical practice remain inconclusive [65]. It has also been shown that there is a possibility of inducing AF in supraventricular arrhythmias. In a study by Wang Z et al., ivabradine increased the risk of developing AF by 23% [66]. However, it was also reported that there was no increase in the incidence of new cases of AF [57]. Although there is no consensus regarding the onset of AF, it has been reported that ivabradine is ineffective for HFrEF accompanied by paroxysmal AF [67]. Therefore, strict monitoring is required at least after initiating ivabradine treatment. Ivabradine has effects on the sinus node, and it has been reported that the use of ivabradine in HF with AF reduces heart rate [68], and it is hoped that the effects of ivabradine on arrhythmias will become clear.

Bradycardia has also been reported as an adverse effect of ivabradine. In the SHIFT study, symptomatic bradycardia was observed in approximately 5% of patients [17], whereas in the J-SHIFT study, no symptomatic bradycardia was observed, and asymptomatic bradycardia was observed in 0.8% of patients [23]. Although these results may be due to differences in the patient backgrounds of the SHIFT and J-SHIFT studies, it has also been reported that the long-term blockade of If channels prevented excessive bradycardia caused by acute vagus nerve activation in rats [69]. Continuous use in patients with sinus tachycardia also led to the long-term normalization of heart rate without excessive bradycardia or syncope [70]. These results were also seen in patients after heart transplantation, where ivabradine was administered safely for 36 months, suggesting that ivabradine is a drug that can be used safely in the long term [71].

Side effect rates and compliance are sometimes problems in the elderly. However, the incidence of the side effects of ivabradine is not related to age [72]. There are five factors that affect medication compliance, and all factors should be considered in elderly HF patients with low compliance [73]. One of these is the number of times medication is taken. Currently, ivabradine is possible to use in Japan at a dose of twice per day. It has been reported that sustained-release ivabradine hemisulfate, taken once daily, has similar efficacy to ivabradine [74]. It is necessary to understand the indications for ivabradine and provide HF treatment using ivabradine in an appropriate setting.

## 8. Conclusions

Ivabradine has no negative inotropic effects and may benefit certain HF patients, including improving exercise capacity. Photopsia as a side effect has also been reported, and its involvement in arrhythmia has not yet been fully clarified. It is important to use ivabradine depending on the patient’s clinical status.

## Figures and Tables

**Table 1:** The effects of ivabradine in heart failure with preserved ejection fraction.

Study	Number of Subjects	Average Heart Rate (bpm)	Average Follow-Up Period	Induction Rates of β-Blockers	Main Outcomes
Komajda, M. et al., 2017 [26]	179	72	8 years	74%	HR reduction with ivabradine did not improve outcome
Cacciapuoti, F. et al., 2017 [28]	26	81	12 weeks	80%	Ivabradine improved LV diastolic function
Conceição, L.S.R. et al., 2021 [29]	136	N/A	N/A	N/A	Ivabradine did not improve LV diastolic function
Kosmala, W. et al., 2013 [30]	61	72	7 days	57%	Ivabradine increased exercise capacity
Tanaka, H. et al., 2022 [27]	18	85	3 months	50%	Ivabradine did not improve LV diastolic function

N/A: not applicable.

**Table 2:** The effects of ivabradine on exercise tolerance.

Study	Number of Subjects	Average Age (Years)	Underlying Heart Disease	Type of Heart Failure	Main Outcomes
Villacorta, A.S. et al., 2019 [41]	21	Ivabradine: 56.2 Pyridostigmine: 62.6	N/A	HFpEF	Ivabradine improved exercise tolerance and neurohormonal and inflammatory profiles
Kosmala, W. et al., 2013 [30]	61	Ivabradine: 66.5 Placebo: 68.0	N/A	HFpEF	Ivabradine increased exercise capacity left ventricular filling pressure
Sarullo, F.M. et al., 2010 [42]	60	52.7	Ischemic Cardiomyopathy	HFrEF	Ivabradine improved exercise tolerance, gas exchange, functional heart failure class, and quality of life
Pal, N. et al. 2018 [43]	22	74.6	N/A	HFpEF	Ivabradine compared with placebo worsened the change in peak Vo2 in HFpEF patients
Volterrani, M. et al., 2013 [44]	121	Ivabradine: 67.2 Carvedilol: 66.7 Combination: 66.5	Ischemic Cardiomyopathy Ivabradine: 80% Carvedilol: 84% Combination: 79%	HFrEF	Ivabradine alone or in combination with carvedilol is more effective than carvedilol alone in improving exercise tolerance

N/A: not applicable; HFrEF: heart failure with reduced ejection fraction; HFpEF: heart failure with preserved ejection fraction.

## Data Availability

Data sharing does not apply to this manuscript as no new data were generated or analyzed.
